# The genome sequence of the Scarlet Tiger moth, *Callimorpha dominula *(Linnaeus, 1758)

**DOI:** 10.12688/wellcomeopenres.20833.1

**Published:** 2024-02-12

**Authors:** Douglas Boyes, Peter W. H. Holland, University of Oxford and Wytham Woods Genome Acquisition Lab, Darwin Tree of Life Barcoding collective, Wellcome Sanger Institute Tree of Life Management, Samples and Laboratory team, Wellcome Sanger Institute Scientific Operations: Sequencing Operations, Wellcome Sanger Institute Tree of Life Core Informatics team, Tree of Life Core Informatics collective, Darwin Tree of Life Consortium

**Affiliations:** 1 UK Centre for Ecology & Hydrology, Wallingford, England, UK; 2 University of Oxford, Oxford, England, UK

**Keywords:** Callimorpha dominula, Scarlet Tiger moth, genome sequence, chromosomal, Lepidoptera

## Abstract

We present a genome assembly from an individual male *Callimorpha dominula* (the Scarlet Tiger moth; Arthropoda; Insecta; Lepidoptera; Erebidae). The genome sequence is 658.1 megabases in span. Most of the assembly is scaffolded into 31 chromosomal pseudomolecules, including the Z sex chromosome. The mitochondrial genome has also been assembled and is 15.45 kilobases in length. Gene annotation of this assembly on Ensembl identified 20,234 protein coding genes.

## Species taxonomy

Eukaryota; Opisthokonta; Metazoa; Eumetazoa; Bilateria; Protostomia; Ecdysozoa; Panarthropoda; Arthropoda; Mandibulata; Pancrustacea; Hexapoda; Insecta; Dicondylia; Pterygota; Neoptera; Endopterygota; Amphiesmenoptera; Lepidoptera; Glossata; Neolepidoptera; Heteroneura; Ditrysia; Obtectomera; Noctuoidea; Erebidae; Arctiinae; Callimorphini; *Callimorpha*; *Callimorpha dominula* (Linnaeus, 1758) (NCBI:txid938182).

## Background

The Scarlet Tiger *Callimorpha dominula* is a day-flying moth with blue-black forewings marked with cream and white spots, and striking red and black hindwings. Several colour variants have been described including the form *bimacula* with only two forewing spots, proposed to result from a homozygous recessive mutation, and a partially spotted form *medionigra* proposed to be heterozygous at the same locus ( Ford, 1967). The *bimacula* allele may also have pleiotropic effects on other traits ( Sheppard & Cook, 1962). *C. dominula* inhabits damp meadows and riverine habitats where the larval foodplant comfrey, *Symphytum officinale*, grows. The distribution in Britain is very patchy, with many apparently suitable habitats not favoured by the species. Where present, however, the moth can be abundant, with the weakly flying adults a common sight in June in some riverside towns in the south and west of England and Wales. The moth is found across most of Europe from southern Scandinavia to northern Spain, and also further east to Iran and Russia ( GBIF Secretariat, 2023).

For several decades, the Scarlet Tiger moth *C. dominula* was at the centre of a fierce academic dispute around the relative importance of natural selection and genetic drift in evolution. From the 1930s to the 1960s, E.B. Ford, Ronald Fisher and Philip Sheppard monitored the frequencies of the normal colour variant, the homozygote *bimacula* and the heterozygote *medionigra* at Cothill Fen near Oxford, UK. They argued that radically changing genotype frequencies seen from year to year, in a relatively large population, could only be explained by changing selection pressures and not by genetic drift ( Fisher & Ford, 1947; Ford, 1967). There have been criticisms of these studies, including statistical critique and evidence for variable penetrance of alleles leading to inconsistent genotype inference by observers ( Clarke *et al.*, 1991; Cook & Jones, 1996). One environmental cause of variable penetrance may be the temperature experienced by the larvae or pupae ( Owen & Goulson, 1994), although the relevance of this effect has also been disputed ( Jones, 2000). Current evidence suggests that natural selection, genetic drift and dispersal were all interacting in the Cothill population ( O’Hara, 2005).

The genome sequence of *Callimorpha dominula* was determined as part of the Darwin Tree of Life project. The complete genome sequence will aid research into the molecular basis of wing colour polymorphisms and facilitate research into habitat choice and larval food preference.

## Genome sequence report

**Figure 1.**
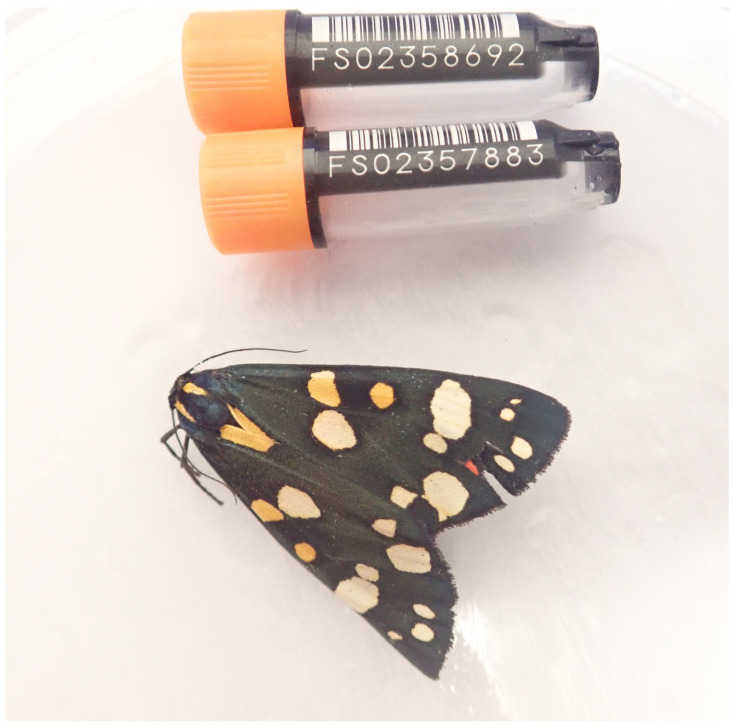
Photograph of the *Callimorpha dominula* (ilCalDomi2) specimen used for genome sequencing.

The genome was sequenced from one male *Callimorpha dominula* ( Figure 1) collected from Wytham Woods, Oxfordshire, UK (51.77, –1.33). A total of 39-fold coverage in Pacific Biosciences single-molecule HiFi long reads was generated. Primary assembly contigs were scaffolded with chromosome conformation Hi-C data. Manual assembly curation corrected 12 missing joins or mis-joins and removed one haplotypic duplication, reducing the scaffold number by 10.81%.

**Figure 2.**
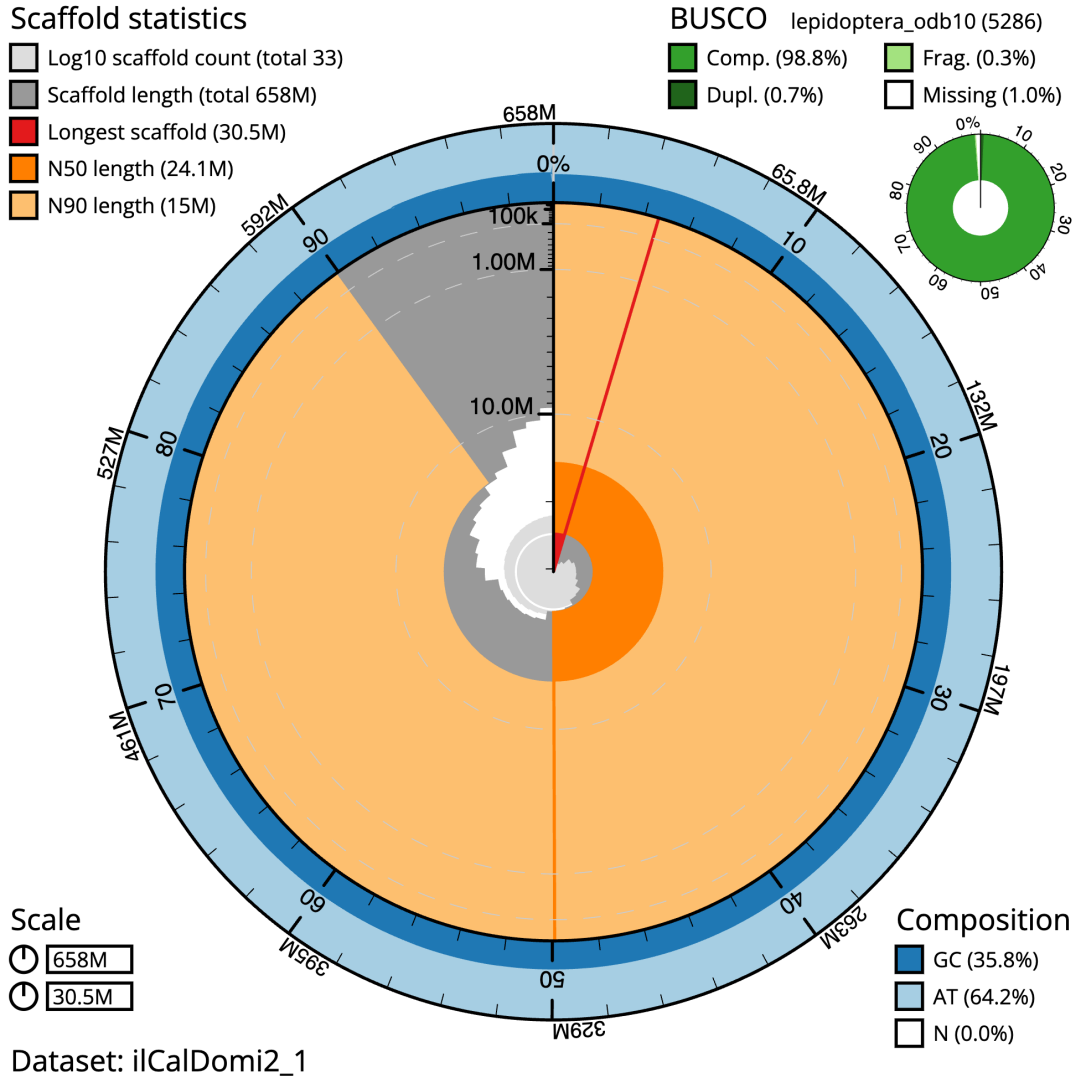
Genome assembly of *Callimorpha dominula*, ilCalDomi2.1: metrics. The BlobToolKit Snailplot shows N50 metrics and BUSCO gene completeness. The main plot is divided into 1,000 size-ordered bins around the circumference with each bin representing 0.1% of the 658,140,099 bp assembly. The distribution of scaffold lengths is shown in dark grey with the plot radius scaled to the longest scaffold present in the assembly (30,463,512 bp, shown in red). Orange and pale-orange arcs show the N50 and N90 scaffold lengths (24,103,837 and 15,029,384 bp), respectively. The pale grey spiral shows the cumulative scaffold count on a log scale with white scale lines showing successive orders of magnitude. The blue and pale-blue area around the outside of the plot shows the distribution of GC, AT and N percentages in the same bins as the inner plot. A summary of complete, fragmented, duplicated and missing BUSCO genes in the lepidoptera_odb10 set is shown in the top right. An interactive version of this figure is available at https://blobtoolkit.genomehubs.org/view/ilCalDomi2_1/dataset/ilCalDomi2_1/snail.

**Figure 3.**
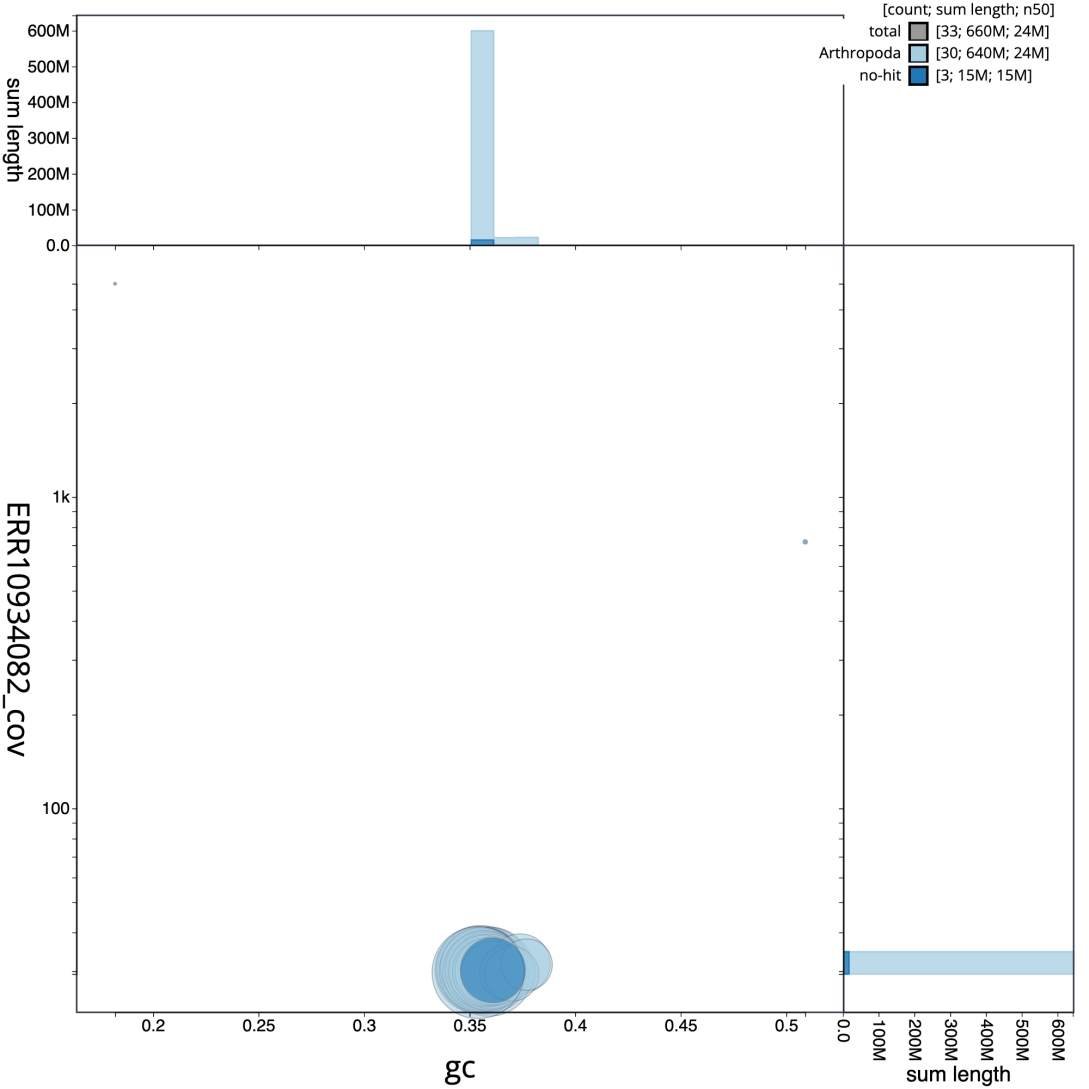
Genome assembly of *Callimorpha dominula*, ilCalDomi2.1: BlobToolKit GC-coverage plot. Scaffolds are coloured by phylum. Circles are sized in proportion to scaffold length. Histograms show the distribution of scaffold length sum along each axis. An interactive version of this figure is available at https://blobtoolkit.genomehubs.org/view/ilCalDomi2_1/dataset/ilCalDomi2_1/blob.

**Figure 4.**
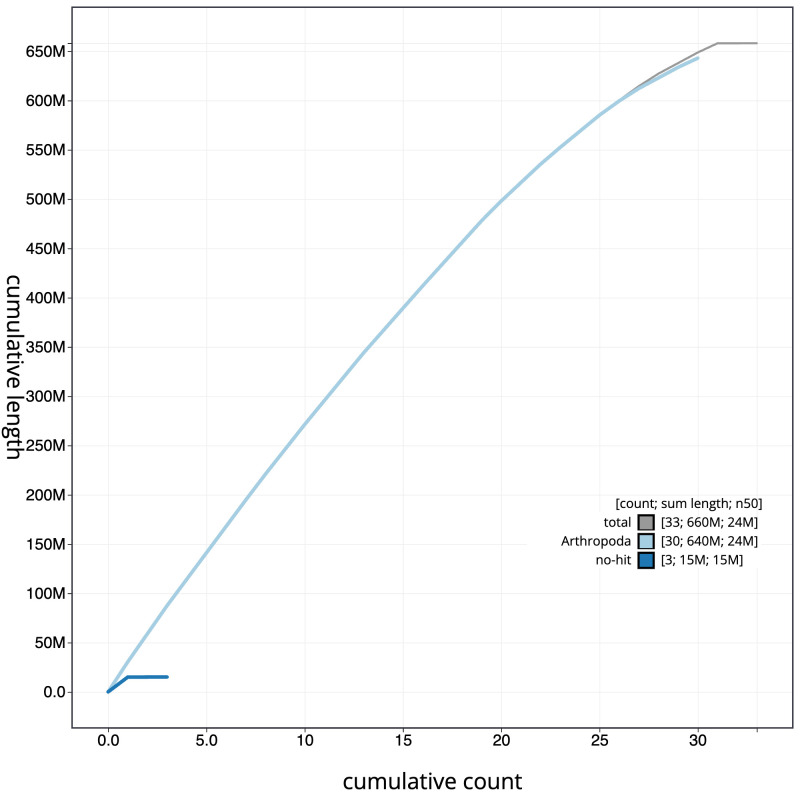
Genome assembly of *Callimorpha dominula*, ilCalDomi2.1: BlobToolKit cumulative sequence plot. The grey line shows cumulative length for all scaffolds. Coloured lines show cumulative lengths of scaffolds assigned to each phylum using the buscogenes taxrule. An interactive version of this figure is available at https://blobtoolkit.genomehubs.org/view/ilCalDomi2_1/dataset/ilCalDomi2_1/cumulative.

**Figure 5.**
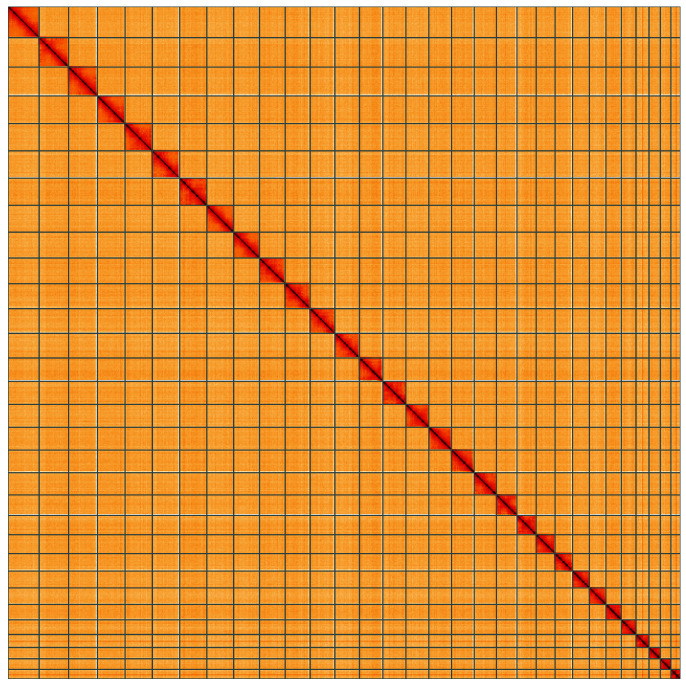
Genome assembly of *Callimorpha dominula*, ilCalDomi2.1: Hi-C contact map of the ilCalDomi2.1 assembly, visualised using HiGlass. Chromosomes are shown in order of size from left to right and top to bottom. An interactive version of this figure may be viewed at https://genome-note-higlass.tol.sanger.ac.uk/l/?d=bE1ClM-NQxG8RLymMc8O3w.

The final assembly has a total length of 658.1 Mb in 32 sequence scaffolds with a scaffold N50 of 24.1 Mb ( Table 1). The snailplot in Figure 2 provides a summary of the assembly statistics, while the distribution of assembly scaffolds on GC proportion and coverage is shown in Figure 3. The cumulative assembly plot in Figure 4 shows curves for subsets of scaffolds assigned to different phyla. Most (99.99%) of the assembly sequence was assigned to 31 chromosomal-level scaffolds, representing 30 autosomes and the Z sex chromosome. Chromosome-scale scaffolds confirmed by the Hi-C data are named in order of size ( Figure 5; Table 2). Chromosome Z has been assigned based on synteny to *Tyria jacobaea* (GCA_947561695.1) ( Boyes *et al.*, 2023). While not fully phased, the assembly deposited is of one haplotype. Contigs corresponding to the second haplotype have also been deposited. The mitochondrial genome was also assembled and can be found as a contig within the multifasta file of the genome submission.

**Table 1.** Genome data for *Callimorpha dominula*, ilCalDomi2.1.

Project accession data		
Assembly identifier	ilCalDomi2.1	
Species	*Callimorpha dominula*	
Specimen	ilCalDomi2	
NCBI taxonomy ID	938182	
BioProject	PRJEB60214	
BioSample ID	SAMEA7701310	
Isolate information	ilCalDomi2, male: head and thorax (DNA and Hi-C sequencing), abdomen (RNA sequencing)	
Assembly metrics *		*Benchmark*
Consensus quality (QV)	65.7	*≥ 50*
*k*-mer completeness	100.0%	*≥ 95%*
BUSCO **	C:98.8%[S:98.1%,D:0.7%], F:0.3%,M:1.0%,n:5,286	*C ≥ 95%*
Percentage of assembly mapped to chromosomes	99.99%	*≥ 95%*
Sex chromosomes	Z	*localised * *homologous pairs*
Organelles	Mitochondrial genome: 15.45 kb	*complete single * *alleles*
Raw data accessions		
PacificBiosciences SEQUEL II	ERR10934082	
Hi-C Illumina	ERR10936425	
PolyA RNA-Seq Illumina	ERR12245540	
Genome assembly		
Assembly accession	GCA_949752705.1	
*Accession of alternate * *haplotype*	GCA_949752685.1	
Span (Mb)	658.1	
Number of contigs	119	
Contig N50 length (Mb)	9.4	
Number of scaffolds	32	
Scaffold N50 length (Mb)	24.1	
Longest scaffold (Mb)	30.46	
Genome annotation		
Number of protein-coding genes	20,234	
Number of gene transcripts	20,422	

* Assembly metric benchmarks are adapted from column VGP-2020 of “Table 1: Proposed standards and metrics for defining genome assembly quality” from ( Rhie *et al.*, 2021). ** BUSCO scores based on the lepidoptera_odb10 BUSCO set using version 5.3.2. C = complete [S = single copy, D = duplicated], F = fragmented, M = missing, n = number of orthologues in comparison. A full set of BUSCO scores is available at https://blobtoolkit.genomehubs.org/view/ilCalDomi2_1/dataset/ilCalDomi2_1/busco.

**Table 2.** Chromosomal pseudomolecules in the genome assembly of *Callimorpha dominula*, ilCalDomi2.

INSDC accession	Chromosome	Length (Mb)	GC%
OX456991.1	1	28.78	36.0
OX456992.1	2	28.24	35.5
OX456993.1	3	27.11	36.0
OX456994.1	4	26.71	35.5
OX456995.1	5	26.69	35.5
OX456996.1	6	26.62	35.5
OX456997.1	7	26.2	35.5
OX456998.1	8	25.32	35.5
OX456999.1	9	25.16	35.5
OX457000.1	10	24.39	35.5
OX457001.1	11	24.23	35.5
OX457002.1	12	24.1	35.5
OX457003.1	13	22.81	35.5
OX457004.1	14	22.61	35.5
OX457005.1	15	22.42	35.5
OX457006.1	16	22.28	36.0
OX457007.1	17	22.06	35.5
OX457008.1	18	21.8	36.0
OX457009.1	19	20.04	36.0
OX457010.1	20	18.88	36.0
OX457011.1	21	18.49	35.5
OX457012.1	22	17.09	36.0
OX457013.1	23	16.57	36.0
OX457014.1	24	16.15	36.0
OX457015.1	25	15.03	36.0
OX457016.1	26	14.37	36.0
OX457017.1	27	12.69	37.5
OX457018.1	28	11.02	37.0
OX457019.1	29	10.44	37.0
OX457020.1	30	9.32	37.5
OX456990.1	Z	30.46	35.5
OX457021.1	MT	0.02	18.5

The estimated Quality Value (QV) of the final assembly is 65.7 with *k*-mer completeness of 100.0%, and the assembly has a BUSCO v5.3.2 completeness of 98.8% (single = 98.1%, duplicated = 0.7%), using the lepidoptera_odb10 reference set ( *n* = 5,286).

Metadata for specimens, barcode results, spectra estimates, sequencing runs, contaminants and pre-curation assembly statistics are given at https://links.tol.sanger.ac.uk/species/938182.

## Genome annotation report

The *Callimorpha dominula* genome assembly (GCA_949752705.1) was annotated using the Ensembl rapid annotation pipeline ( Table 1; https://rapid.ensembl.org/Callimorpha_dominula_GCA_949752705.1/Info/Index). The resulting annotation includes 20,422 transcribed mRNAs from 20,234 protein-coding genes.

## Methods

### Sample acquisition and nucleic acid extraction

A male *Callimorpha dominula* (specimen ID Ox000543, ToLID ilCalDomi2) was collected from Wytham Woods, Oxfordshire, UK (latitude 51.77, longitude –1.33) on 2020-06-25 using a light trap. The specimen was collected and identified by Douglas Boyes (University of Oxford) and preserved on dry ice.

The workflow for high molecular weight (HMW) DNA extraction at the Wellcome Sanger Institute (WSI) includes a sequence of core procedures: sample preparation; sample homogenisation, DNA extraction, fragmentation, and clean-up. In sample preparation, the ilCalDomi2 sample was weighed and dissected on dry ice ( Jay *et al.*, 2023). Tissue from the head and thorax was homogenised using a PowerMasher II tissue disruptor ( Denton *et al.*, 2023a). HMW DNA was extracted in the WSI Scientific Operations core using the Automated MagAttract v2 protocol ( Oatley *et al.*, 2023). The DNA was sheared into an average fragment size of 12–20 kb in a Megaruptor 3 system with speed setting 31 ( Bates *et al.*, 2023). Sheared DNA was purified by solid-phase reversible immobilisation ( Strickland *et al.*, 2023): in brief, the method employs a 1.8X ratio of AMPure PB beads to sample to eliminate shorter fragments and concentrate the DNA. The concentration of the sheared and purified DNA was assessed using a Nanodrop spectrophotometer and Qubit Fluorometer and Qubit dsDNA High Sensitivity Assay kit. Fragment size distribution was evaluated by running the sample on the FemtoPulse system.

RNA was extracted from abdomen tissue of ilCalDomi2 in the Tree of Life Laboratory at the WSI using the RNA Extraction: Automated MagMax™ *mir*Vana protocol ( do Amaral *et al.*, 2023). The RNA concentration was assessed using a Nanodrop spectrophotometer and a Qubit Fluorometer using the Qubit RNA Broad-Range Assay kit. Analysis of the integrity of the RNA was done using the Agilent RNA 6000 Pico Kit and Eukaryotic Total RNA assay.

Protocols developed by the WSI Tree of Life laboratory are publicly available on protocols.io ( Denton *et al.*, 2023b).

### Sequencing

Pacific Biosciences HiFi circular consensus DNA sequencing libraries were constructed according to the manufacturers’ instructions. Poly(A) RNA-Seq libraries were constructed using the NEB Ultra II RNA Library Prep kit. DNA and RNA sequencing was performed by the Scientific Operations core at the WSI on Pacific Biosciences SEQUEL II (HiFi) and Illumina NovaSeq 6000 (RNA-Seq) instruments. Hi-C data were also generated from remaining head and thorax tissue of ilCalDomi2 using the Arima2 kit and sequenced on the Illumina NovaSeq 6000 instrument.

### Genome assembly, curation and evaluation

Assembly was carried out with Hifiasm ( Cheng *et al.*, 2021) and haplotypic duplication was identified and removed with purge_dups ( Guan *et al.*, 2020). The assembly was then scaffolded with Hi-C data ( Rao *et al.*, 2014) using YaHS ( Zhou *et al.*, 2023). The assembly was checked for contamination and corrected using the TreeVal pipeline ( Pointon *et al.*, 2023). Manual curation was performed using JBrowse2 ( Diesh *et al.*, 2023), HiGlass ( Kerpedjiev *et al.*, 2018) and Pretext ( Harry, 2022). The mitochondrial genome was assembled using MitoHiFi ( Uliano-Silva *et al.*, 2023), which runs MitoFinder ( Allio *et al.*, 2020) or MITOS ( Bernt *et al.*, 2013) and uses these annotations to select the final mitochondrial contig and to ensure the general quality of the sequence.

A Hi-C map for the final assembly was produced using bwa-mem2 ( Vasimuddin *et al.*, 2019) in the Cooler file format ( Abdennur & Mirny, 2020). To assess the assembly metrics, the *k*-mer completeness and QV consensus quality values were calculated in Merqury ( Rhie *et al.*, 2020). This work was done using Nextflow ( Di Tommaso *et al.*, 2017) DSL2 pipelines “sanger-tol/readmapping” ( Surana *et al.*, 2023a) and “sanger-tol/genomenote” ( Surana *et al.*, 2023b). The genome was analysed within the BlobToolKit environment ( Challis *et al.*, 2020) and BUSCO scores ( Manni *et al.*, 2021; Simão *et al.*, 2015) were calculated.

Table 3 contains a list of relevant software tool versions and sources.

**Table 3.** Software tools: versions and sources.

Software tool	Version	Source
BlobToolKit	4.2.1	https://github.com/blobtoolkit/blobtoolkit
BUSCO	5.3.2	https://gitlab.com/ezlab/busco
Hifiasm	0.16.1-r375	https://github.com/chhylp123/hifiasm
HiGlass	1.11.6	https://github.com/higlass/higlass
Merqury	MerquryFK	https://github.com/thegenemyers/MERQURY.FK
MitoHiFi	3	https://github.com/marcelauliano/MitoHiFi
PretextView	0.2	https://github.com/wtsi-hpag/PretextView
purge_dups	1.2.3	https://github.com/dfguan/purge_dups
sanger-tol/ genomenote	v1.0	https://github.com/sanger-tol/genomenote
sanger-tol/ readmapping	1.1.0	https://github.com/sanger-tol/readmapping/tree/1.1.0
TreeVal	-	https://github.com/sanger-tol/treeval
YaHS	1.2a	https://github.com/c-zhou/yahs

### Genome annotation

The BRAKER2 pipeline ( Brůna *et al.*, 2021) was used in the default protein mode to generate annotation for the *Callimorpha dominula* assembly (GCA_949752705.1) in Ensembl Rapid Release.

### Wellcome Sanger Institute – Legal and Governance

The materials that have contributed to this genome note have been supplied by a Darwin Tree of Life Partner. The submission of materials by a Darwin Tree of Life Partner is subject to the **‘Darwin Tree of Life Project Sampling Code of Practice’**, which can be found in full on the Darwin Tree of Life website here. By agreeing with and signing up to the Sampling Code of Practice, the Darwin Tree of Life Partner agrees they will meet the legal and ethical requirements and standards set out within this document in respect of all samples acquired for, and supplied to, the Darwin Tree of Life Project. 

Further, the Wellcome Sanger Institute employs a process whereby due diligence is carried out proportionate to the nature of the materials themselves, and the circumstances under which they have been/are to be collected and provided for use. The purpose of this is to address and mitigate any potential legal and/or ethical implications of receipt and use of the materials as part of the research project, and to ensure that in doing so we align with best practice wherever possible. The overarching areas of consideration are:

• Ethical review of provenance and sourcing of the material

• Legality of collection, transfer and use (national and international) 

Each transfer of samples is further undertaken according to a Research Collaboration Agreement or Material Transfer Agreement entered into by the Darwin Tree of Life Partner, Genome Research Limited (operating as the Wellcome Sanger Institute), and in some circumstances other Darwin Tree of Life collaborators.

## Data Availability

European Nucleotide Archive: *Callimorpha dominula* (scarlet tiger). Accession number PRJEB60214; https://identifiers.org/ena.embl/PRJEB60214 ( Wellcome Sanger Institute, 2023). The genome sequence is released openly for reuse. The *Callimorpha dominula* genome sequencing initiative is part of the Darwin Tree of Life (DToL) project. All raw sequence data and the assembly have been deposited in INSDC databases. Raw data and assembly accession identifiers are reported in Table 1.

## Author information

Members of the University of Oxford and Wytham Woods Genome Acquisition Lab are listed here: https://doi.org/10.5281/zenodo.7125292.

Members of the Darwin Tree of Life Barcoding collective are listed here: https://doi.org/10.5281/zenodo.4893703.

Members of the Wellcome Sanger Institute Tree of Life Management, Samples and Laboratory team are listed here: https://doi.org/10.5281/zenodo.10066175.

Members of Wellcome Sanger Institute Scientific Operations: Sequencing Operations are listed here: https://doi.org/10.5281/zenodo.10043364.

Members of the Wellcome Sanger Institute Tree of Life Core Informatics team are listed here: https://doi.org/10.5281/zenodo.10066637.

Members of the Tree of Life Core Informatics collective are listed here: https://doi.org/10.5281/zenodo.5013541.

Members of the Darwin Tree of Life Consortium are listed here: https://doi.org/10.5281/zenodo.4783558.
