# Supplementary material for: The genome sequence of the Scarlet Tiger moth, Callimorpha dominula (Linnaeus, 1758)
Source: Wellcome Open Res. 2024 Feb 12;9:31. [Version 1] doi: 10.12688/wellcomeopenres.20833.1 (PMC11372354; doi:10.12688/wellcomeopenres.20833.1)
Supplement: Peer review history

# Reviewer report: Reviewer response for version 1

**DOI:** 10.21956/wellcomeopenres.23052.r91679

**Published:** 2024-10-21

**Authors:** Arun Arumugaperumal

**Affiliations:** 1 Department of Biotechnology, Rajalakshmi Engineering College, Chennai, Tamilnadu 602105, India

The data note presents the whole genome sequence of *Callimorpha dominula* commonly known as the Tiger moth. This is the first report of the whole genome of the insect. The genome was found to contain 20,234 protein coding genes. The genome size was reported as 658.1 Mb consisting of 31 chromosomes including Z chromosome. 

 The specimen was nicely photographed and presented as figure 1. However a scale could have been included in the photograph.

 The genome sequencing procedure was followed as mentioned in a standard protocol. The data links provided were working and in accessible form. Further work could be done utilizing this huge amount of molecular sequence data.

Are sufficient details of methods and materials provided to allow replication by others?

Yes

Is the rationale for creating the dataset(s) clearly described?

Yes

Are the datasets clearly presented in a useable and accessible format?

Yes

Are the protocols appropriate and is the work technically sound?

Yes

Reviewer Expertise:

Bioinformatics, Genomics

I confirm that I have read this submission and believe that I have an appropriate level of expertise to confirm that it is of an acceptable scientific standard.

# Reviewer report: Reviewer response for version 1

**DOI:** 10.21956/wellcomeopenres.23052.r96842

**Published:** 2024-09-03

**Authors:** Juan Wulff

**Affiliations:** 1 North Carolina State University, Raleigh, USA

**Abstract: **please add the word of in between as follows … “a male individual” of “ *Callimorpha dominula*”…

**Abstract:** since genetic annotation is one of the weaknesses of this work I recommend to the authors removing the sentence “Gene annotation of this assembly on Ensembl identified 20,234 protein coding genes.” from it.

 Please rephrase to clarify if “ *bimacula*” is another species or just a variant, *e.g*. “Several colour variants of the same species”

 For future reports, using a scale bar in the pictures could be useful to see the actual specimens’ size.

**Keywords:** chromosomal is not a word that provides more visualization, or a strong keyword associated with the study; given the background, a word more related to the evolution of the species or those factors that drive the evolution of the species might be a better choice.

 It may be useful for future studies if the DNA of bacteria and other microorganisms is provided separately from the genome assembly but all together with an associated ID within the same Bioproject.

 It is recommended to use a transcriptomic approach to improve gene annotation in future studies.

Are sufficient details of methods and materials provided to allow replication by others?

Yes

Is the rationale for creating the dataset(s) clearly described?

Yes

Are the datasets clearly presented in a useable and accessible format?

Yes

Are the protocols appropriate and is the work technically sound?

Yes

Reviewer Expertise:

Entomology; Molecular Biology; Genetics

I confirm that I have read this submission and believe that I have an appropriate level of expertise to confirm that it is of an acceptable scientific standard.

# Reviewer report: Reviewer response for version 1

**DOI:** 10.21956/wellcomeopenres.23052.r83182

**Published:** 2024-06-13

**Authors:** Alena Sucháčková Bartonova

**Affiliations:** 1 Institute of Entomology, Biology Centre of the Czech Academy of Sciences, České Budějovice, Czech Republic

The article represents yet another Lepidopteran genome assembly, this time of *Callimorpha dominula*, including mitogenome assembly and gene annotation. The manuscript is standard and the text is clear. The data are stored in a standard way.

 I have only one minor text change suggestion:

 Background -

- Specify the range: further east to NW Iran and Ural Mountains.

Are sufficient details of methods and materials provided to allow replication by others?

Yes

Is the rationale for creating the dataset(s) clearly described?

Yes

Are the datasets clearly presented in a useable and accessible format?

Yes

Are the protocols appropriate and is the work technically sound?

Yes

Reviewer Expertise:

biogeography, population genetics, phylogeography, Lepidopteran ecology

I confirm that I have read this submission and believe that I have an appropriate level of expertise to confirm that it is of an acceptable scientific standard.
